# Why are family carers of people with dementia dissatisfied with general hospital care? a qualitative study

**DOI:** 10.1186/1471-2318-12-57

**Published:** 2012-09-24

**Authors:** Fiona J Jurgens, Philip Clissett, John RF Gladman, Rowan H Harwood

**Affiliations:** 1 Division of Rehabilitation and Ageing, University of Nottingham, Nottingham, NG7 2UH, UK; 2 School of Nursing, University of Nottingham, Nottingham, NG7 2UH, UK; 3 Health Care for Older People, Nottingham University Hospitals NHS Trust, Queens Medical Centre, Nottingham, NG7 2UH, UK

**Keywords:** Aged, Acute hospital care, Dementia, Delirium, Family carers, Satisfaction with care, Carer strain, Qualitative study, United Kingdom

## Abstract

**Background:**

Families and other carers report widespread dissatisfaction with general hospital care for confused older people.

**Methods:**

We undertook a qualitative interviews study of 35 family carers of 34 confused older patients to ascertain their experiences of care on geriatric and general medical, and orthopaedic wards of a large English hospital. Transcripts were analysed using a grounded theory approach. Themes identified in interviews were categorised, and used to build a model explaining dissatisfaction with care.

**Results:**

The experience of hospital care was often negative. Key themes were events (illness leading to admission, experiences in the hospital, adverse occurrences including deterioration in health, or perceived poor care); expectations (which were sometimes unrealistic, usually unexplored by staff, and largely unmet from the carers’ perspective); and relationships with staff (poor communication and conflict over care). Expectations were influenced by prior experience. A cycle of discontent is proposed. Events (or ‘crises’) are associated with expectations. When these are unmet, carers become uncertain or suspicious, which leads to a period of ‘hyper vigilant monitoring’ during which carers seek out evidence of poor care, culminating in challenge, conflict with staff, or withdrawal, itself a crisis. The cycle could be completed early during the admission pathway, and multiple cycles within a single admission were seen.

**Conclusion:**

People with dementia who have family carers should be considered together as a unit. Family carers are often stressed and tired, and need engaging and reassuring. They need to give and receive information about the care of the person with dementia, and offered the opportunity to participate in care whilst in hospital. Understanding the perspective of the family carer, and recognising elements of the ‘cycle of discontent’, could help ward staff anticipate carer needs, enable relationship building, to pre-empt or avoid dissatisfaction or conflict.

## Background

People with dementia are prone to develop acute physical illnesses leading to hospital admission. About 40% of people over the age of 65 in general hospitals have delirium, dementia or both together [1,2]. Families and other carers report widespread dissatisfaction with hospital care for such people [3]. However, there has been little systematic study of their views [4].

We report part of an ethnographic study of older people with mental health problems admitted to medical or trauma orthopaedic wards as an emergency, and their family carers [5]. This paper describes an analysis of interviews with family carers conducted after discharge, and presents a model which aims to explain dissatisfaction.

## Methods

We screened patients over 70 years of age for mental health problems, including cognitive impairment, depression, alcoholism or evidence of other psychiatric disorder. Patients were admitted as an emergency to one of 12 general or geriatric medical, or trauma orthopaedic wards on two sites of a large English teaching hospital, providing sole emergency hospital services for a population of 660 000. Two hundred and fifty patients (‘patient participants’), thought likely to have a mental health problem on the basis of screening, were recruited to a follow up study [2]. Patients with capacity gave their own written, informed, consent. Consultee agreement for participation was sought from a family carer if capacity was lacking. Family carers were also invited to take part (‘carer participants’), and gave their own consent, including to be approached for an interview. A carer was defined as someone having at least weekly contact with the patient participant.

An observational and interview study was conducted, using non-participant observation on hospital wards, and interviewing family carers (and patients, if possible) after discharge. An ethnographic approach, taking the perspective of the patient was used. Traditional ethnography is a branch of anthropology dealing with the scientific description of culture [6]. The group studied here was hospitalised older people with mental health problems, predominantly delirium and dementia. The culture under study was revealed through the interactions between the confused older person, family carers, other patients who shared the ward (co-patients), and the various types of staff who cared for them.

Seventy-two hours of non-participant observations of care were performed on the study wards. The original intention had been to interview carers of people whose care had been observed on the ward, but this proved difficult to arrange in practice. A convenience sub-sample of carers of the 250 patient-participants was therefore asked by telephone to take part in an interview on their experiences of hospital care, six to eight weeks after discharge (12 weeks if the patient had died). Recruitment continued until saturation of data occurred (no new ideas were emerging during analysis). Thirty-five interviews about 34 patients were conducted [5].

Interviews took place in the patient’s or carer’s home or care home, by one of two experienced post-doctoral, qualitative researchers (FJ, PC), one male, one female, both of whom had a nursing background, but not involved in clinical care. Most interviews were performed with the patient and carer together, some separately, with most of the information coming from the carer-participant. In four cases several family members wished to be present together. On one occasion a second interview was undertaken with an additional family carer. A semi-structured schedule of topics (see Appendix) was used to guide interviews, but this was not rigidly adhered to, and lines of questioning developed as the study progressed, initial data were discussed, and coding themes emerged. Interviews lasted on average one hour (range 20 minutes to two hours). They were audio recorded, transcribed, and anonymised before analysis, and pseudonyms were allocated for identification. Field notes were made.

We used a grounded theory approach to analysis [7]. Data were managed using N-Vivo 8 software (QRS International Pty). Coding was by three researchers (FJ, PC and a professor of nursing who supervised the analyses), using the constant comparative method. Themes were identified, and their meaning discussed to achieve consensus. During this process it was noticed that there was a subset of data which highlighted how family carers who experienced dissatisfaction engaged in particular patterns of activity. From this a model was constructed to describe and explain these data.

### Role of the funding source

The funder and sponsor had no role in study design, or collection, analysis, or interpretation of data, writing the report, or the decision to submit for publication.

## Results

### Participants

Of the 250 patients recruited to the follow-up study, 195 (79%) were cognitively impaired. Ninety-six family carers were approached for interviews and 35 agreed, relating to 34 patient participants. Thirty-one of the patient participants had dementia, with or without super-imposed delirium, one a learning disability, and two other mental health problems. For convenience, we have followed the common British general hospital practice of referring to these patients as ‘confused’, whilst conceding that the term is vague, and strictly only applies to those with delirium and dementia.

Reasons for non-participation included readmission, fatigue, carer being too ill, family disagreements, other caring responsibilities, and paid work. Those with no family carer were excluded. It proved very difficult to recruit those with depression alone.

The mean age of the patient participants was 87 years (range 70–99); 19 (56%) were female; 21 (62%) were widowed, nine (26%) were married, two (6%) had never married, and two (6%) were divorced. Sixteen had previously lived alone, of whom six returned, eight were discharged to a care home, and two died. Eleven had previously lived with family, of who five returned, four went to a care home, and two died. Seven had previously lived in a care home, three died in hospital, the rest returned. A further five patients died between discharge and the time of the interview.

The relationship of 32 of the carers to the patient was recorded: nine spouses, eight daughters, seven sons, two nieces, two female friends, two sisters, one son-in-law, and one grand-daughter. The mean age of carers was 63 (range 46–79), and 24 (69%) were female.

Interviews with several family members present differed from those involving only a single carer. Accounts were sometimes challenged by other family members as they were given. Interviews also differed when the person with dementia was present, with examples of jocularity and stoicism, but also with family members excluding the person with dementia from the conversation, using infantile language, talking over, disregarding and teasing.

### Hospital admission

#### The admission process

Family carers reported that caring for a relative with dementia is demanding, with little time for rest. In the period leading up to an acute admission, the person with dementia frequently became more difficult to manage because of disorientation, challenging behaviours and reduced physical function. At the point of hospitalisation the family carer was often physically and emotionally exhausted.

Family carers had previously coped with the challenges of being with a person with dementia, and described satisfaction in doing so. Hospital admission inevitably led to disruption of caring routines and was experienced as a ‘crisis’ by the family carer regardless of the quality of care given by health care professionals. Hospital admissions did not provide ‘respite’: carers found travelling and adaptations to their routine stressful.

"“I just said that I couldn’t do it any more … I said, ‘you cannot be rushing down there [hospital], not getting any sleep, the following day you’re down there again’” [Bernie, daughter of April]."

Some carers praised the job done by paramedics. However, all gave negative accounts of the admission experience. Typically, this focused around transportation, and the hospital Emergency Department. Based on previous experience, some families sought to delay admission, worrying that the process would be more harmful than the physical illness.

"“she wasn’t agitated when she was at home but as soon as the medics started getting her into the ambulance, she went berserk. They couldn’t give her a sedative and, apparently, they can’t physically restrain them now.” [Brian, son of Hannah]."

All participants described at least one event that led to dissatisfaction, and these usually occurred early in the admission:

"“they were too busy. But with a person that’s mentally impaired you can’t control them, and she was trying to get off the trolley…she hadn’t even had anything for the pain at that time” [Sally, daughter of Victoria]."

#### The ward

Family carers’ experiences were variable, in all types of ward. Carers’ concerns early in the admission were about symptoms and their cause, resisting early discharge, bed moves, and lack of communication with professionals. Later on, concern focused on the delivery or appropriateness of interventions and, frequently, deterioration in the health of the person with dementia. Unexpected changes in the condition of the person with dementia were often attributed to the effects of hospital care. Family carers were surprised at failure to provide for the particular needs of people with dementia, or to ask family members for information:

"“I would have thought with dad having dementia that the first time somebody went onto that ward, when he’d just gone on it there was a system in place where they came and asked you things, because they must know as well as we know that dementia patients doesn’t remember things, gets things mixed up … But nobody ever came to talk to us.” [Tina, daughter of Eric]."

Quality of care was judged in terms of food, hydration, maintaining safety, showing warmth towards patient and family carer, using appropriate approaches to caring for a confused person, and suitable medical care. Soiled sheets, insertion of urinary catheters, physical isolation and loneliness, and cracked lips were viewed as indicators of poor care. The ability to manage agitation and pain were used by carers to judge the competence of health professionals. Family carers thought staff were most concerned with delivering medical treatment and task-orientated care. Explanations by staff were not always believed.

"“And so I got lots of fluids down her then, but of course the more fluids I’m going to get down her the more is going to come out, you only pee that stuff, and so I’d be telling them she’s drank a lot, she’s going to need changing, and then I’d be worried, are they going to change her? Because apparently if they’ve changed her once it don’t need doing again until the afternoon, well that’s not the way it works.” [Sally, daughter of Victoria]."

Not all carers wanted to give active physical and emotional care to the person with dementia during the hospitalisation, but some assumed they had to. Conversely, some family carers wanted to continue to actively care for the person with dementia throughout the hospital stay. When doing so they questioned the staff’s training, attitudes towards older people, and accountability. Staff were thought to look young and inexperienced, and were felt to avoid older patients.

"“I think the young ones are a bit scared of them because I don’t think they know how to handle them…maybe you have to have special, if you’re going in for mental nursing maybe that’s when they teach them, but if you’re just doing a general nursing maybe they don’t.” [Linda, wife of Roger]."

Most family carers felt they had to act as advocates for the person with dementia. Some did not feel willing or capable of making decisions about care, especially regarding feeding tubes, intravenous infusions, or resuscitation. They expected health professionals to make these decisions and interpreted discussions about such decisions as an additional pressure.

#### Discharge

In the most positive accounts, discharge followed deliberation and planning. Other accounts of discharge were characterised by poor planning and lack of consultation.

"“Well, it would have been nice, well… to have been told that he was coming out, then I could have been up at the flat, even if I couldn’t have got in the flat, I could have been there for when he come home. Then I could have done him a dinner, made sure he’d got everything he needed. But nobody told me so I didn’t know.” [Paula, neighbour of Derek]."

In one case re-admission was within 12 hours of discharge and this provoked anger:

"“to kick somebody 88 year old and she wasn’t right even when she came out, to kick her out at half past ten at night to me that’s not right. Especially when you’re not happy yourself about it you know, I still wasn’t convinced that she was right, you know, when they let her out” [Ken, son of Maria]."

End-of-life care also provoked tensions. Several accounts were positive, but where end-of-life care was thought sub-standard, carers were left with a sense of injustice, personal blame and anger.

### Key themes

Throughout the interviews, family carers described ‘crises’, meaning crucial moments, turning-points, times of distress or emergencies. Six foci were identified: the person with dementia; family carers; communication with professionals, organisation of the hospital; treatment; and the influence of family and friends (Table 1). These interacted in three further themes: expectations, events and relationships.

**Table 1:** Themes emerging from interviews with family carers of people with dementia

**Category**	**Theme**
The person with dementia	Physical and mental health problems
	Stage of dementia
	Distress behaviours
	Previously expressed wishes
	Current perceptions and wishes
	Quality of life
Family carers	Physical and mental health problems
	Exhaustion
	Own experiences of hospital
	Judgements of benefit of admission and treatment
	Relationship with the person with dementia
	Relationship with healthcare professionals
Communication with professionals	Level of trust
	Accurate information
	Ease and amount of individualised contact
	Judgment of professionalism
	Attitudes towards older people and those with dementia
	Convergence in expectations about outcomes
Treatment	Medical and nursing care
	Basic hygiene needs
	Feeding and cups of tea
	Comfort needs and timely response
	Number of invasive procedures and necessity for them
	Medication, sedation and pain control
	Restraint
	Multiple bed moves
	End-of-life transitions
Family and friends	Geography of family
	Ease of access to hospital
	Advocacy network
	Tensions between members of family and friends
	Charities for support
Systems	Bed moves and ward transfers
	‘Four hour wait’ target in emergency department*
	Signage in emergency department
	Visiting times
	Treatment and advocacy policies

* Government policy that patients attending the emergency department should be assessed, treated and discharged or admitted to a ward within four hours of arrival.

#### Expectations

Unmet expectations regarding care were a consistent source of concern. These expectations were often about maintaining dignity, physical comfort, ensuring privacy, identity, and safety. It was expected that hospitals would have systems in place to manage people with disturbed behaviour and would be a place of safety.

Past experiences, and the family carers’ knowledge of the person with dementia, were important in shaping expectations about hospital admissions. Every carer used comparisons with previous experiences such as care in nursing homes, or their own or family members’ previous hospital admissions. Family carers expected a personalized style of care in hospital, which contrasted with the busy experience of an acute hospital ward.

#### Events

Events that could trigger the experience of a crisis were frequent and included the change in health of the person with dementia before admission, the admission process itself, and unexpected changes after admission.

"“Well he completely changed when he was in there, completely. It sent him even more wappy [crazy] than what he was when he went in. He wouldn’t let you touch anything, if you went anywhere near his clothes or anything he’d scream at you, LEAVE THEM ALONE, THEY’RE MINE”. [Martha wife of Ralph]."

#### Relationship breakdown

On occasions, relationship breakdown between patients, family carers and health professionals occurred. For example, family carers who wished to contribute towards care in hospital were sometimes prevented from doing so, without explanation. Family carers recalled hospital staff deliberately ignoring them, being patronising or inflexible. Information that staff collected tended to be directed towards discharge rather than towards better care. Most carers felt that they could not approach staff to volunteer information. Poor communication was rationalised as an indication of stressed and inexperienced staff, but lack of information caused anger and frustration. Eventually, staff could be blamed for poor outcomes and further events:

"“But we just feel that, because of what happened in the hospital, it wasn’t the dementia that was taking her life, it was her knee, her knee caused her to die, you know, she, dementia doesn’t, you don’t die of dementia like that” [Sally, daughter of Flo]."

### The cycle of discontent

**Figure 1:**
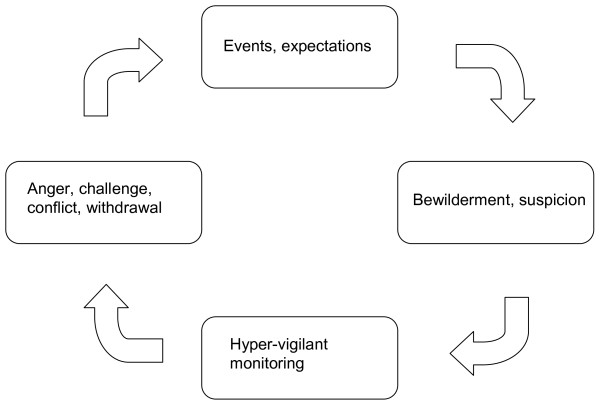
The cycle of discontent.

We propose a ‘cycle of discontent’, a pattern of responses shown by family carers when they felt concerns about the quality of care, and loss of control. The model has four phases: events and expectations; bewilderment and suspicion; evidence gathering, and anger and conflict. It is illustrated in Figure 1.

If a carer notices something of concern, a period of uncertainty and suspicion follows if they cannot explain it. If concerns are not allayed, the family carer can respond by actively monitoring care and interactions on the ward, and gathering further evidence. Such ‘hyper-vigilant monitoring’ includes arriving on the ward unannounced outside visiting hours, questioning other visitors and patients, checking on hygiene around the bed area, seeking internet information on the quality of hospital care, and (surreptitiously) scrutinising charts and nursing notes.

Feelings of mistrust and anger increasingly develop if concerns are confirmed. Family carers can become confrontational, or withdraw from staff. By this stage communication with staff is considered to be futile and emotions run high. Since staff by this stage may be regarded as unprofessional and not to be trusted, concerns are often vented beyond ward boundaries and include consultation with advocacy groups, other professionals such as general practitioners, formal complaints, and in extreme cases removal of the patient from the ward.

The cycle could be completed early during the admission pathway, and multiple cycles within a single admission were seen.

## Discussion

Family carers’ experiences of general hospital care for people with dementia vary, but for some it is a time of bewilderment, worry, disappointment, and conflict. Family carers were critical of staff expertise in dealing with problems such as agitation or disturbed behaviour, of poor awareness and attitudes towards delirium and dementia, and problems such as communication difficulties. Many carers are left with feelings of anger and resentment, which they carry from one admission to the next. This can lead to a ‘cycle of discontent’, explained in terms of prior expectations, events that take place before and during a hospital stay, and breakdown of relationships.

The data are limited by coming from 12 wards across the two sites of a single English National Health Service hospital organisation, although experiences appeared to be common across locations. Interviews were done some weeks after hospitalisation, which might influence perceptions and interpretations of experiences, and which aspects of the story were related to the interviewer. Family carers were often interviewed with the patient participant present, which might limit what was said openly. Patient participants contributed to interviews on some occasions, and the interviewers discussed some aspects separately from the patient if the family carer indicated this was desirable. Analysis of qualitative data is open to different interpretations and the possibility of preconception. Against this, wide-ranging semi-structured interviews can uncover areas of concern for participants that might not be anticipated in advance. Our study cannot answer whether family carers’ perceptions of hospitals are accurate or fair. Rather, we report the way some family carers are thinking. We have focussed on why family carers can become dissatisfied or angry, rather than attempting a more comprehensive explanation of family carers experiences of hospital care, which we report elsewhere [8].

There has been little previous systematic study of carers’ experiences, including how families perceive the process of hospitalisation, and decisions about care [4,9], despite this issue being recognised as key to providing appropriate care for people with dementia [10]. Most studies have been small, and describe staff opinions. Reports include distress caused by communication failings, staff not understanding dementia [3,11], lack of opportunity to share knowledge about the person with dementia, or to participate fully in decision making and discharge planning, and discrepancies about perceived standards of care between health professionals and family carers [4,9]. The relationship between family members and staff can include mutual wariness; relatives can be afraid to air their concerns for fear of reprisals against the patient, and staff can be afraid to communicate with relatives lest they be held responsible for failures in care [12]. However, family carers report valuing the work done by professionals [13], and can be recognised as assets by healthcare staff, because of their potential to facilitate care [14]. Others have stressed the importance of staff recognising the needs of family carers as well as the patient, and the need to develop a therapeutic relationship between staff and family as well as with the patient [15].

There is a vast literature on patient satisfaction and complaints [16]. Little of it is devoted to patients with dementia [17]. Literature that has considered hospitalisation and the needs of relatives more generally, has highlighted the disruption to family members associated with a prolonged hospital stay [18], the need to work out ‘hospital rules’ and then follow them [19], and the feelings of disempowerment that can arise from the experience [20]. Family members also made judgements about the quality of care [21], the ability and suitability of individual members of staff [22], and sometimes worried about what might be happening in the hospital when they were not there [23].

The cycle of discontent outlined in this paper offers an insight into how these issues can combine in a negative way resulting in family carer dissatisfaction. The early phases of the cycle of discontent have similarities to Expectation Confirmation Theory, a marketing theory originally developed by Oliver [24]. This suggests that consumers of goods and services go through a number of processes in deciding on their level of satisfaction: they hold prior expectations; reach judgements about the quality of the good or service; the perception of quality either confirms or fails to confirm (disconfirms) these expectations; and the consumer reaches a judgement regarding their satisfaction with the service [25]. Within Expectation Confirmation Theory, it is anticipated that consumers will use their evaluation of satisfaction to inform future purchasing decisions [24]. For many carers of people with dementia, admission to hospital cannot be avoided for their relatives, meaning that they are trapped in the ‘purchase’ of a service with which they are dissatisfied, leading to the later stages of the cycle of discontent, and the reported attempts to avert or delay hospital admission.

Understanding the concerns of family carers, how they might be thinking, and why they are acting the way they do, may help staff to avoid or defuse problems. For example, when family carers challenge staff, it may mean that they feel that their relative is in danger. A defensive or confrontational response contributes to further relationship breakdown and exacerbates the discontent. Early identification and intervention based on proactive and empathetic discussion, which focuses on the expectations of family carers, and aiming to agree a shared narrative of the cause of the crisis might reduce dissatisfaction, facilitate the building of relationships and sustain ongoing support. In the cycle we describe, the phases of uncertainty and evidence gathering represented distinctive patterns which professionals should learn to recognise and respond to, by immediate effort to inform and gain the trust of the family carers.

Care of confused older people should be multi-disciplinary, but much attention focuses on nursing. The main model of nursing practice in Britain, Roper’s model, does not explicitly include cognition, relationships, or understanding behaviour from the patient’s perspective [26]. The model implicitly focuses on physical problems, and on the individual rather than relationships. Alternative models have developed notions of ‘person-centred’ and ‘relationship-centred’ care, within the patient-staff-family carer triad [27-29].

Adapting these models to the needs of the acute hospital is challenging, and needs further development. Research is required to identify aspects of care that improve carer satisfaction with hospital care, and interventions that systematically attempt to improve care for both patients and their family carers [30,31]. Healthcare commissioners and providers should give greater emphasis to the inclusion and experience of family carers of people with dementia, as is more commonly seen in child health and palliative care services.

## Conclusions

Instead of a simple patient-professional relationship, the experience of confused older people in hospital involves a ‘triad’, also including family carers. Their experience of hospital care is variable, but often negative. These experiences are driven by events, expectations, and perceived poor communication with staff, and are often interpreted in relation to previous contact with health services. Expectations can be unrealistic, but are usually unexplored by staff, and therefore unmet. We identified a characteristic series of stages. Events left carers anxious and stressed. Failure of services to meet expectations led to suspicion, ‘hypervigilant monitoring’, and subsequently to conflict or withdrawal. We propose that recognising these stages can help staff pre-empt problems, and better address patient and family carer needs through inclusion and proactive communication.

## Ethical approval

The study was approved by the Bradford Research Ethics Committee (08HC005).

## Competing interests

The authors declare that they have no competing interests.

## Authors’ contributions

JG and RH conceived the study. Interviews were undertaken by FJ and PC. FJ, and PC undertook the literature search and analysed the data. FJ wrote the initial draft, which was revised by RH and JG. All authors contributed to editing**,** and approved the final version.

## Appendix

### Interview Schedule: Family and Carers version

1. Introduction.

2. Admission: what bought them into hospital.

3. Being in Hospital.

4. Expectations of hospitalisation.

5. What helped and what did not help regarding hospitalisation.

6. Discharge arrangements.

7. Expectations of discharge arrangements.

8. What helped and what did not help regarding discharge.

9. Resettlement at home.

10. Expectations on return to home from hospital.

11. What helped and what did not help regarding resettlement from hospital.

12. Transfer to Community services (if applicable).

13. Expectations of transfer to community services from hospital.

14. What helped and what did not help regarding transfer from hospital.

15. Ongoing issues/problems related to hospitalisation episode.

1. Introduction

I am a researcher from the University of Nottingham.

I am here to discuss with you the recent hospitalisation of …(name) in ..(state hospital).

I am very sorry to hear of your recent loss (for those recently bereaved).

(Reiterate consent, confidentiality and that interviewee may withdraw at any time).

This interview will be taped and last about one hour.

2. Admission

Tell me about [name]…

Prompts:

Why was [name] in hospital?

Were you with [name] when they went to hospital?

Who informed you?

What facilities were there for you at the hospital? (prompts: time of day/distance/ambulance and so on).

Is there anything that would have made it better?

What sort of support did you receive from the hospital?

Can you tell me any good things about the care your family and [name] received?

What more could have been done to help you?

3. Being in hospital

How long was [name] in hospital? Did you manage to see [name] whilst in hospital?

Was this as often as you wanted? If not why not? Explore issues to see if hospital derived.

Do you think this was successful?

Were you informed about [name]s condition whilst in hospital? If so who told you and how?

4. Expectations of hospitalisation

What did you expect to happen whilst [name] was in hospital?

Did you let any member of staff know about your expectations for [name]?

Can you tell me more about that?

5. What helped and what didn’t help regarding being in hospital

Thinking about the time in hospital…

Can you tell me any good things about the care you and [name] received?

Can you tell me any less than good care that you think [name] received?

Did you receive poor care yourself?

What more could have been done to help you?

When you think what happened, do you think anything would have made it easier?

6.Discharge Arrangements

Can you tell me when you were first made aware of plans for discharge?

How were you made aware?

Can you tell me more about that?

7. Expectations of discharge arrangements

What sort of discharge did you expect?

Were you surprised?

(give prompts about medication, meals on wheels and so on if necessary).

8. What helped and what didn’t help discharge

Thinking about the discharge arrangements and actual discharge:

Can you think of anything the hospital could have done to make it easier?

Can you tell me any good things about the care you and [name] received about the discharge?

Can you tell me any poor things about the care you and [name] received about the discharge?

9. Resettlement

Tell me about how [name] has settled since leaving hospital?

What sort of support did you receive from the hospital?

Did [name]experience any problems which could have prevented by the hospital?

Can you tell me more about that?

10. Expectations

Tell me your expectations for [name] when he arrived home?

Do you think these have been realised?

If not, why not?

Have they been exceeded? If so can you tell me more?

11.What helped and what didn’t help resettlement

Thinking only about resettlement:

Can you tell me any good things about the care in hospital which you think directly helped the resettlement back into home for [name]?

Can you think of anything which about the care in hospital which you think directly hindered the resettlement back into the home for [name]?

Can you suggest anything the hospital could have done to make resettlement easier for [name]?

12./13./14. As for resettlement but specific to residential home/nursing home.

15. Ongoing problems

Is there anything else you would like to share with me about your experience?

**Additional probes** will be used to elicit more information about each topic as appropriate. Such as:

Then what happened?

Can you tell me more?

You said..what do you mean by that?

Can you explain that a little more?

Have you been helped by anyone else in the hospital?

How did you find out about that?

Is there anything that would have made it better?

What would have really helped at that time?

What would have made a difference?

Bereaved Family and carers

Adapt interview to focus on being in hospital and issues according to families/carers pace & direction of issues.

## Pre-publication history

The pre-publication history for this paper can be accessed here:

http://www.biomedcentral.com/1471-2318/12/57/prepub
